# The decline of ‘Deaths of Despair’ in Italy: unveiling this phenomenon in a new context

**DOI:** 10.1186/s12963-025-00430-9

**Published:** 2026-01-09

**Authors:** Giacomo Lanfiuti Baldi, Andrea Nigri, Sergi Trias-Llimós, Elisabetta Barbi

**Affiliations:** 1 Department of Statistics, Sapienza University of Rome, Rome, Italy; 2 Department of Social Sciences, University of Foggia, Foggia, Italy; 3 Department of Political and Social Sciences, Pompeu Fabra University, Barcelona, Spain

**Keywords:** Deaths-of-Despair, Causes-of-Deaths, Mortality, Italy, Alcohol, Drugs, Suicide

## Abstract

**Background:**

The term “Deaths of Despair” (DoD) refers to mortality due to alcohol consumption, drug use, and suicides. While extensively studied in the United States, where these deaths have markedly increased, less is known about their patterns in other contexts. This study explores the relevance of this concept to Italy, a country with comparatively lower rates, to determine whether these causes of death exhibit common trends and can be meaningfully grouped under a single category.

**Methods:**

We use cause-specific mortality data from the Italian National Institute of Statistics covering the period 1983 to 2018. Data are aggregated by gender and five-year age groups at the NUTS1 regional level. The Potential Gain in Life Expectancy is used to assess the impact of each cause on overall mortality. To explore potential long-term relationships between the causes and across regions, we apply Cointegration Analysis to the time series.

**Results:**

The analysis shows a general decline in mortality from causes typically associated with despair in Italy, mainly driven by a reduction in alcohol-related deaths. Drug-related mortality and suicide show more heterogeneous trends across regions and over time. Cointegration Analysis reveals no evidence of long-term dependency among these causes or across regions, with only a few exceptions. This indicates that the observed causes do not share a common underlying temporal structure.

**Conclusions:**

Findings suggest that in the Italian context, deaths from alcohol, drugs, and suicide do not follow a unified pattern and should not be treated as a single category. Rather, they represent distinct public health issues with different regional trajectories and determinants. As such, they require targeted and differentiated policy responses rather than a unified approach.

## Introduction

In the global longevity scenario, Italy is within the countries competing for leading positions in the evolution of life expectancy [40]. Over the 20th century, the life expectancy at birth of Italian women has risen from 50 to 84.7 years, with an average increase of 2.9 years per decade [26].

According to [38], Italian public health policy could promote future increases in life expectancy by focusing on reducing cancer and cardiovascular diseases (CVD) for both sexes and mortality from external causes for men. The reduction in cardiovascular mortality has made the most significant contributions to improvements in life expectancy for middle-aged and older adults. Causes of death have become increasingly diverse in recent decades as a result of gains in life expectancy, with potential consequences for healthcare systems. The authors note that in specific periods, for both sexes, improvements in life expectancy were primarily due to reductions in mortality from digestive and respiratory diseases, as well as decreased mortality at very young ages (0 to 5 years). While these causes remain central for guiding public health interventions, other emerging causes of death have received comparatively less attention, despite their potential relevance for understanding recent trends in overall mortality.

More recently, global focus has shifted away from the primary causes of mortality, directing attention instead towards the phenomenon known as "Deaths of Despair" [14]. This category is often treated as a unified category encompassing three distinct causes: mortality attributable to alcohol consumption, drug abuse, and suicide. These factors have been identified as contributing significantly to the increasing mortality rates among white non-Hispanic middle-aged individuals in the United States [15], where this phenomenon has been predominantly studied. Nevertheless, the mechanisms by which these trends potentially occur, behave, and evolve in other countries remain less well-known, and frequently neglected in research.

Italy is among the countries with the lowest despair-related mortality rates [33] and, to the best of our knowledge, no study has investigated Deaths of Despair as a whole within the country. Some studies, mainly in official statistics reports, have dealt with the three causes individually in Italy. A country where the regional socio-economic gradient is well known [12, 37] and this is also reflected in inequalities in longevity and mortality [9, 23, 34] across the country, specifically for certain causes of death [8, 19]. About Deaths of Despair, regional disparities in alcohol- and suicide-related mortality are evident in Italy, while drug-related mortality remains less thoroughly studied. Suicide rates are predominantly higher in the northern regions of the country and are more frequently recorded among men [47]. The economic crisis that originated in 2008 marked a turning point, with a notable upward shift in suicide trends [42, 48]. Over the past decades, alcohol consumption in Italy has declined and remains below the European average [28]. Alcohol-related mortality has similarly decreased, although significant regional differences persist [27]. In contrast to suicide trends, alcohol-related mortality demonstrates an inverse regional gradient, with higher rates reported in southern Italy [27].

In this context, tthe present study aims to assess mortality trends related to despair in Italy, using a macroarea-level analysis. Examining Italy is valuable because it allows us to observe deaths of despair within a context marked by very low mortality, unique regional socio-economic disparities and distinct cultural attitudes toward self-destructive behaviours. Specifically, we leverage a double lens of the potential gain in life expectancy approach combined with the time series analysis to discover potential trends and impending changes in Death of Despair evolution. Thus, our primary objective is to evaluate the impact of Deaths of Despair on the overall mortality in Italy and to investigate both the joint and independent dynamics of these causes of death.

This approach has the potential to provide policymakers with a quantifiable measure of the phenomenon and to expand understanding of its trajectory within a new country and setting, beyond the contexts traditionally explored in existing literature. Specifically, it is crucial to assess whether in Italy Deaths of Despair should be approached as a single group of causes of death or if each component merits independent intervention to effectively reduce its impact.

In Section "State of art on Deaths of Despair" we provide an overview of the topic Deaths of Despair: where it arose, in which countries it was addressed and what results were found. Section "Data" contains information on the data and codes used to select the causes of death. Section "Methods" is devoted to explaining the methods used in the analyses. In Sect. "Results" we set out the results we found with the national and regional analyses. Finally, Section "Discussion" discusses the results and concludes the paper. Appendices A, B, C, and D respectively contain the Italian map with Nuts1 classification, distributions of Age-at-Death of despair, further results of the analyses at the Nuts1 level, and other results obtained using different measures.

## State of art on Deaths of Despair

The term Deaths of Despair was coined by [14] and further explored in subsequent papers in [15] and [16]. This term reflects the assumption, supported by evidence—particularly in the United States—that deaths from suicide, alcohol-related causes, and drug overdoses are associated with self-destructive behaviors driven by a sense of despair. A dramatic increase in mortality from these causes has been documented, with shared determinants such as economic hardship, social disconnection, and declining life satisfaction contributing to this trend [15, 16]. The authors emphasized an alarming rise in mortality rates among middle-aged non-Hispanic whites since the late 1990 s, and in [16], they described the ongoing crisis as an "Epidemic of Despair." Furthermore, regional patterns have emerged as a crucial aspect of Deaths of Despair research, with significant geographic disparities observed in the prevalence of these deaths and their underlying causes [44]. Three major high-risk clusters arose across the U.S. from 2000 to 2019, in Appalachia, the western U.S., and the southeastern U.S. These areas, which experience overlapping issues of overdose, suicide, and liver disease, underscore the importance of addressing shared risk factors and vulnerabilities across different types of deaths of despair in specific regions.

While the term Deaths of Despair has often been used in reference to the US population as a whole, the original formulation by [14] was explicitly focused on white non-Hispanic middle-aged individuals, particularly men, and especially those with low educational attainment. This specificity is crucial: even within the United States, subsequent studies have shown that the simultaneous increase in suicide, alcohol-related, and drug-related mortality is not uniformly observed across all demographic groups. Later academic and media coverage sometimes extended the concept to other causes of death or broader populations, but empirical evidence for such generalisation remains mixed.

Outside the US, studies on trends in Deaths of Despair are limited but growing. For instance, [2] reported that Scotland shows a similar trend to the US, particularly with a significant increase in drug-related mortality. In other countries, the situation is less severe, and therefore the topic has received less attention. The rest of the UK and Canada compared with the US and Scotland did not observe such dramatic increases in deaths of despair [20]. However, there has been an upward trend in drug-related deaths and suicides. Irrespective of the recent time trends, a recent study in England found significant cross-regional differences in deaths of despair, suggesting notable differences exist even within a country [11].

To broaden the discussion beyond English-speaking countries, [31] compared the US with Eastern European countries. They observed a similar health crisis linked to the rapid transition from socialism to capitalism. This transition led to an unprecedented increase in mortality, particularly among middle-aged men, due to economic dislocation, stress, and despair.

High despair-related death rates have been observed in countries like South Korea, mirroring trends in the US. Other high-income nations, including Australia and Sweden, report slightly lower but still significant rates. In contrast, Southern European countries such as Italy, Spain, and Turkey experience a relatively lower impact of these causes on overall population health [33]. Additionally, differences within countries exist. For instance, education was found to be positively associated with lower DoD rates [25, 41].

In this context, the present study extends the analysis of mortality trends related to despair in Italy, using a macroarea-level analysis.

## Data

We analyse trends in despair-related mortality in Italy across four decades from 1983 to 2018. The data on deaths and mid-year population estimates come from the Italian National Statistical Institute (ISTAT).

Data are provided at the provincial level. In this study, however, we aggregate them to the five Italian macro-areas (NUTS1) for both substantive and methodological reasons. Substantively, this is, to our knowledge, the first work to provide a systematic assessment of Deaths of Despair in Italy; starting from NUTS1 offers an overall view of the phenomenon and a policy-relevant scale that aligns with the well-documented North–South socio-economic and health divide in Italy [9, 23]. Methodologically, the type of analyses we conduct requires a balance between the number of time series and their smoothness. Aggregation at the NUTS1 level helps reduce small-number volatility (especially for drug-related mortality in narrow age–sex strata) and provides sufficiently stable series over time to allow for meaningful comparisons across macro-regions. In addition, this classification is consistent with the territorial breakdown commonly used in official Italian statistical reports (e.g., [27, 27]), making our results more directly interpretable in a policy context.

NUTS1 represents the major Italian group of regions: North-West (Valle d’Aosta, Lombardia, Piemonte e Liguria), North-East (Veneto, Emilia-Romagna, Friuli Venezia-Giulia and the Autonomous provinces of Bolzano and Trento), Centre (Toscana, Umbria, Marche and Lazio), South (Abruzzo, Campania, Molise, Puglia and Basilicata), and Islands (Sicilia e Sardegna). See Appendix A for the geographical representation on the map of Italy.

The data is grouped into five-year age bands, up to 90+, and all analyses were conducted separately for males and females.

ISTAT data provide information on the leading cause of death, classified according to the ICD-9 system from 1983 to 2002, and the ICD-10 system from 2003 to 2018. For our analysis, we focused on the three specific causes of death associated with despair as highlighted in the literature (Alcohol and Drugs related mortality and Suicides). To select the deaths due to these causes we adopt the ICD codes used by [41]. In particular, we identified deaths related to alcohol as those coded under chronic liver disease and cirrhosis, alcohol poisoning and other alcohol-related disorders. Drug-related deaths encompass mental and behavioral disorders due to drug use as well as accidental poisoning from drug exposure. Lastly, suicide deaths also include cases where the intent is undetermined, acknowledging the challenges in accurately coding such sensitive cases. The corresponding ICD codes for these categories are detailed in Table 1. We caught some slight trend breaks in the transition between ICD9 and ICD10. However slight they are, the presence of trend breaks will be taken into consideration during the analysis of the time series.

**Table 1:** Codes of Deaths of Despair as in Table 1 of [41]

	ICD-9	ICD-10
Alcohol	291, 303, 305.0, 357.5, 425.5, 535.3, 571 (excluding 571.6), E860	F10, K70, K73, X45, G31.2, G62.1, I42.6, K29.2, K74.0, K74.2, K74.6, K86.0
Drugs	292, 304, 305.2–305.9.2.9, E850-E858	F11-F16, F18-F19, X40-X44
Suicide	E950-E959, E980-E989	X60-X84, Y10-Y34, Y87.0, Y87.2

## Methods

### Potential gain in life expectancy

The *Potential Gain in Life Expectancy* (PGLE) provides estimates of the increase in life expectancy at birth if a specific cause of death was eliminated from a population [46]. This measure provides valuable insights into the relative impact of different causes of mortality on overall mortality. In this framework, all the individuals in the population are assumed to be subject to a number of independent competing causes of death acting simultaneously [32]. It is necessary to assume the causes of death as independent.

The number of years gained in life expectancy at birth is calculated by subtracting the usual life expectancy in the presence of all causes of death from that in the corresponding scenario in which one or several causes of death are removed.

Formally, the computation of PGLE is based on the framework of *multiple decrement life tables*, which provide a standard approach for modelling competing causes of death [10, 43]. In this setting, overall mortality rates are decomposed into cause-specific rates, and eliminating a cause corresponds to setting its associated decrements to zero while keeping the others unchanged. This procedure yields a counterfactual survival schedule and life expectancy, allowing us to quantify the gain in years of life at birth if that specific cause were absent.

Then, defining $D_x$ the death count at age *x* due to all causes of death, and $D_x^*=D_x-D_x^C$ the deaths due to all causes of death except the deaths due to the removed causes ($D_x^C$) we can compute two different mortality rates for each specific age ($m_x$ and $m^*_x$), leveraging the same mid-year exposure population ($E_x$). Consequently, two life tables are calculated based on the two mortality rates, each providing a life expectancy at birth: respectively, $e_0$ from all causes of death-life table and $e_0^*$ from the life tables with cause elimination.

The potential gain in life expectancy is defined as:

1 $$\begin{aligned} PGLE=e_0^*-e_o \end{aligned}$$

The Potential Gain in Life Expectancy is, therefore, a quantity measured in years that takes values between 0, if no death is due to one of the removed causes, to infinity, if all causes of death are removed.

The PGLE by construction takes into account the difference between the distribution of the age at death of the removed cause and that of all causes together. Removing deaths occurring at younger ages has a greater impact on life expectancy.

Although conceptually related, PGLE differs from alternative measures such as *Years of Life Lost* (YLL), which quantify the total years lost due to premature mortality. In our study, both indicators were computed and yielded consistent results (see Appendix D). We focus on PGLE in the main text because its expression in terms of gains in life expectancy makes it more directly interpretable and comparable across populations.

### Cointegration analysis

Cointegration Analysis is a tool useful to understand and model the dependence between time series variables [5]. We are interested in better understanding whether the impacts of despair-related causes of death within and between different parts of Italy are co-integrated, and so if there exist long-run equilibrium relationships between them.

We define, for example, a multivariate time series containing the impacts of a single cause of death in different areas for the same years. As defined by [21], the components of a multivariate time series are said to be cointegrated if they have an integration order equal to or higher than 1, and it is possible to arrange at least one linear combination out of them whose integration order is smaller than input series. This means that if the components are integrated of order 1, then they are cointegrated if there is at least one linear combination of them that is stationary [17].

Cointegration analysis has been developed in the field of econometrics, but has recently also gained attention in longevity studies [18] and mortality modelling (e.g. see [1, 39]) and forecasting [13]. Extensive use of cointegration analysis to study dependency links and the long-term equilibrium between causes of death was made by [5] in several works [4–7]. We use a workflow similar to that used in these papers.

Let’s define the multivariate time-series $\varvec{y_t}$, that consists of the *n* Non-Stationary elements $y_{it}$, for $i, \dots , n$. The elements of the multivariate time-series are said to be cointegrated, with a cointegrating vector $\beta$, if a linear combination $\beta '\varvec{y_t}$ is stationary:

$$\begin{aligned} \beta _1 y_{1t} + \beta _2 y_{2t} + \dots + \beta _n y_{nt} = z_t, \end{aligned}$$

where $z_t$ is a stationary variable of stochastic deviations.

To model Non-Stationary, but cointegrating time-series, VECM models were specified:

$$\begin{aligned} \varvec{\Delta y_t} &= \varvec{c} + \varvec{d}t + \varvec{\Gamma _1} \varvec{\Delta y_{t-1}} \\&+ \varvec{\Gamma _2} \varvec{\Delta y_{t-2}} + \dots + \varvec{\Gamma _{p-1}} \varvec{\Delta y_{t-p+1}} + \varvec{\Pi y_{t-1}} + \varvec{\epsilon _t}, \end{aligned}$$

where $\varvec{\Delta y_t} = \varvec{y_t}-\varvec{y_{t-1}}$ denote the first differences of the data time series, $\varvec{c}$ and $\varvec{d}$ are $n \times 1$ vectors of constants, $\varvec{\Gamma _i}$ is a $n \times n$ matrix of autoregressive coefficients for $i, \dots , p-1$, and $\varvec{\epsilon _t}$ is the white noise term of the model. We are interested in $\varvec{\Pi y_{t-1}}$, which represents the cointegrated term and provides the information on the long-run equilibrium between the time series: the rank of the matrix $\varvec{\Pi }$ corresponds to the number of cointegration relations. Thus, if the rank of $\varvec{\Pi }$ - henceforth, *r* - is greater than zero, the process is considered cointegrated. With $r = 0$, on the other hand, the processes $\varvec{y_t}$ are devoid of cointegrating relationships. [30] proposed two tests to identify the number cointegration relations, if they exist, via the trace and the maximum eigenvalues tests on matrix $\varvec{\Pi }$.

We then define the steps to follow in order to define whether multivariate time series are cointegrated: first of all, we check that the time series that make up the multivariate time series are Non-Stationary and therefore present a unit root. We test the hypothesis of the presence of a unit root in the time series with the best-recognised tests: Augmented Dickey-Fuller (ADF) and Phillips-Perron (PP). We also use the complementary Kwiatkowski-Phillips-Schmidt-Shin (KPSS) test in which the null hypothesis is the stationarity of the series. Analogously, but with opposite acceptance regions, we study the stationarity of the time series of first-order differences.

We then apply the Johansen approach to define whether cointegration links between the series are present and in what number. Finally, we test the stationarity of these relationships to ensure that they constitute a long-run dependency structure. All tests performed with critical values of 95%, unless otherwise reported.

## Results

### Decreasing impact of Deaths of Despair

The impact of a specific cause of death on the overall mortality depends on the age at which it occurs. Suicide, Alcohol and Drugs have a different age-at-death distribution from all-cause mortality (see Fig. 4 in Appendix B). Removing deaths occurring at younger ages has a greater impact on the gain in life expectancy.

**Fig. 1:**
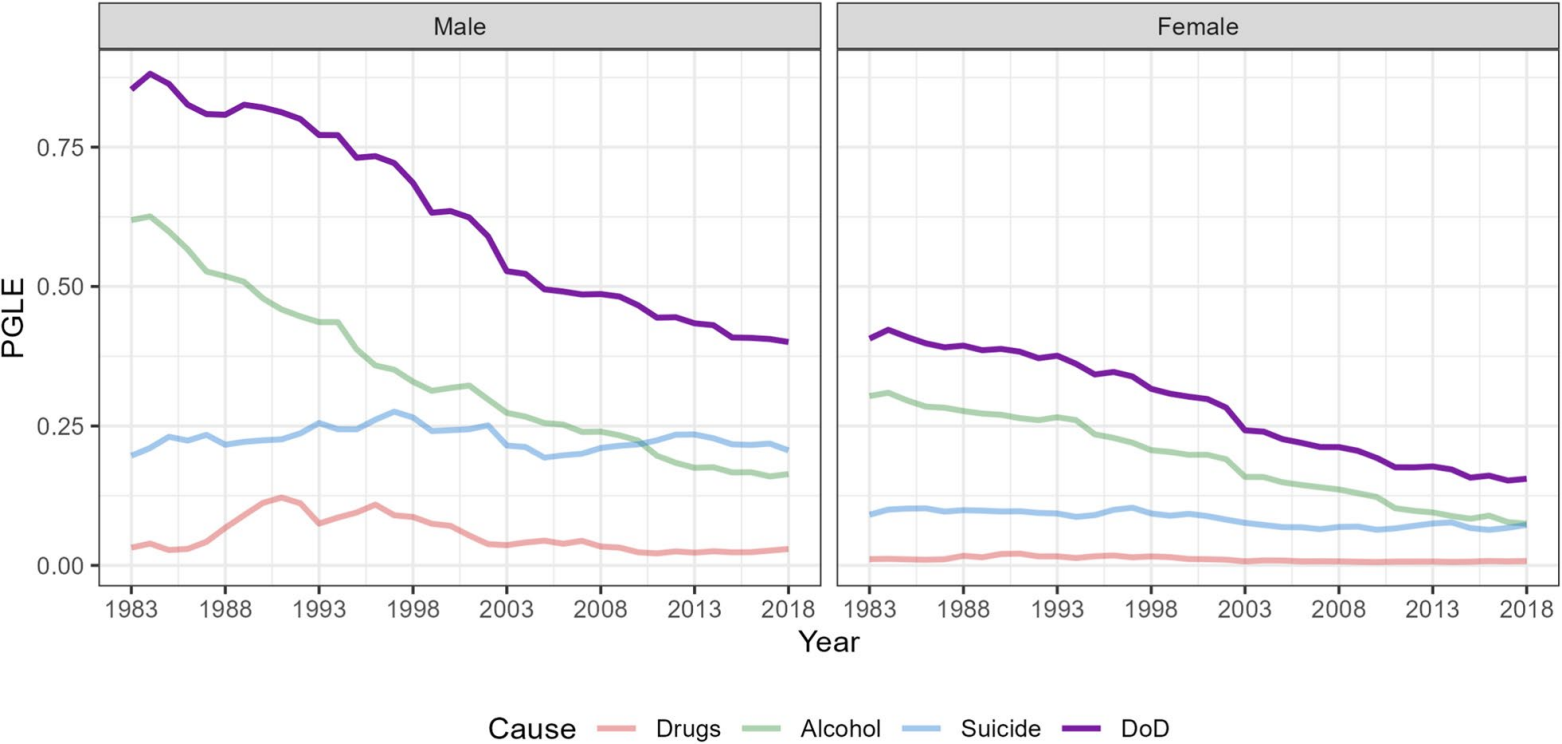
Potential Gain in Life Expectancy removing Drugs, Alcohol and Suicide separately or together (DoD, violet line). Italy 1983-2018

In Italy, the impact of deaths of despair on the population’s mortality fell substantially between 1983 and 2018 see Fig. 1. The PGLE value has more than halved over the period considered: the highest value was in 1984 among men with almost a year of life lost ($PGLE=0.85y$) due to all DoD, while the lowest is in 2018 when we calculated 0.40 years of life expectancy gained. For women, the gain in life expectancy went from 0.42 years in 1984 to 0.16 in 2018.

This decline is driven by alcohol-related mortality: the PGLE by removing this cause alone fell from 0.63y to 0.16y for men, and from 0.31y to 0.07y for women. The impact of suicides and drug-related deaths has remained almost constant over time, leading to a convergence between these three causes of death.

As a consequence of the economic crisis of 2008, in the male population suicides exceeded the impact of alcohol-related deaths in 2010, while among women only in the last year analyzed. In contrast to the evidence from the United States, where drug-related deaths have the highest impact, in Italy these have unremarkable levels compared to the others.

**Fig. 2:**
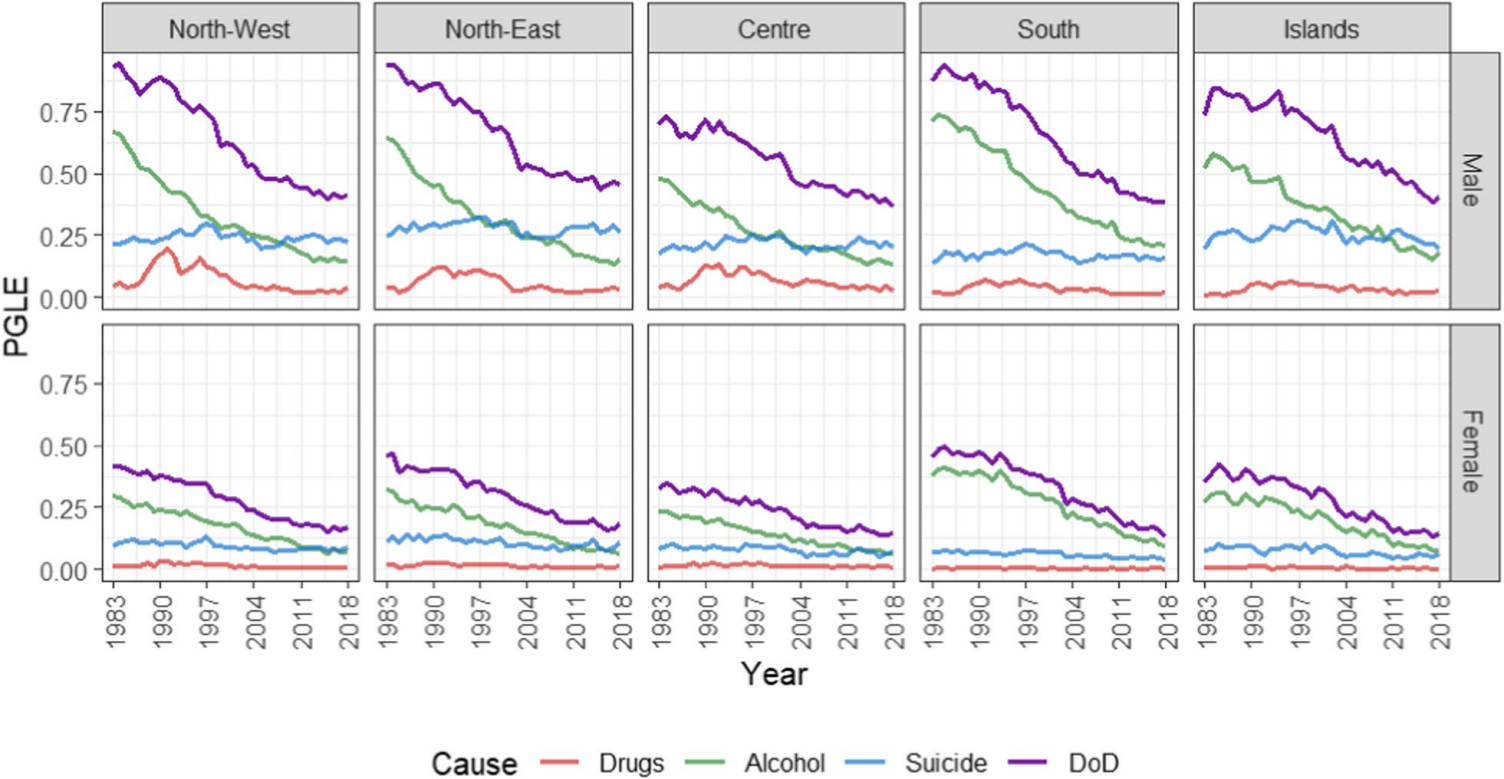
Potential Gain in Life Expectancy removing Drugs, Alcohol and Suicide separately or together (DoD, violet line)

**Fig. 4:**
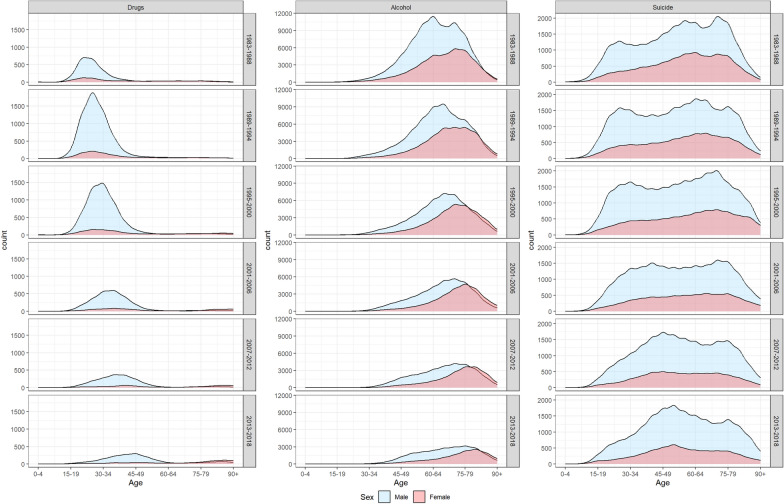
Distribution of age at death for each of the 3 causes within deaths of despair grouped in 5-year intervals. Italy 1983-2018

**Fig. 5:**
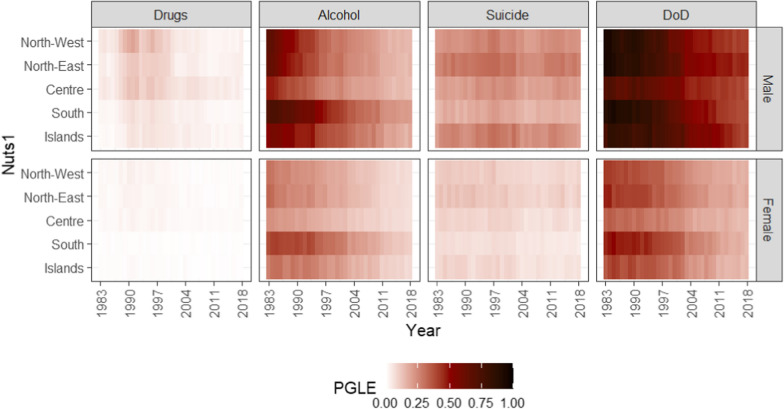
Heat Map of PGLE by causes of deaths separately or together. Darker colors represent higher Potential Gain value. Nuts1 1983-2018

Overall, we observe a similar trend in PGLE by eliminating DoD mortality in all Italian macro-areas, but with some differences in the magnitude of the different causes of death (Fig. 2). See also Fig. 5 in Appendix C for a better understanding of the PGLE trend of the specific causes in the specific areas.

Considering the three aggregated causes (violet line) in the 1980 s, the areas where the impact of deaths of despair is greatest are the northern areas and the South, while in the Islands and especially in the Centre, the burden was less evident. In the last years analysed, we observe a process of convergence, but the North-East area continues to have the highest PGLE values.

More interesting observations arise from the analysis of the individual causes. Drug-related deaths are the least impacting, but we evidence high PGLE values mainly in the North and Centre during the 1990 s (in 1990 for men in the North-West about 0.2 years of PGLE). In the last two decades, the impact of drug-related mortality has declined sharply, remaining higher in the Centre than in other areas.

In contrast, the Centre is the area that historically reports the lowest alcohol-related PGLE values. Concerning this cause, the highest values are observed, during the entire period, in the North (both East and West) and the South. Only in the latter area, however, the impact of alcohol-related mortality is still the highest of the three causes. In the North, Centre and Islands in the last years analysed, Suicide represents the most worrying cause of death (in 2018 for men in the North-East 3 months –0.25 years– of PGLE). The opposite trend between alcohol-related deaths and suicides and the difference between the South and the other areas shows that the two causes are, at least in part, competitive. The reported findings are equally valid for both males and females, keeping in mind, however, that the values in the female population are much lower.

### Cointegration analysis between and within areas

We observed very different trends between the three causes of the Deaths of Despair classification and also saw some differences and some similarities between the trends of the different areas concerning the individual causes. To identify whether a dependency structure exists in the Italian context between the causes that make up this new classification we use Cointegration Analysis as explained in the Sect. "Cointegration analysis".

We are interested in finding out whether there is a long-run equilibrium between the three causes, which would indicate a common trend and thus a common reaction to "variations in despair" in Italy, and a long-run equilibrium between different areas of the country in the trend of each cause, which would indicate a homogeneous response of the country to "variations in despair".

We then indicate two types of analysis:

- **Within**-area: investigates the dependency structure of the three causes *within* each area,
- **Between**-areas: investigates the equilibrium of trends *between* each region concerning each cause.

We perform Cointegration Analysis on the time-series of PGLEs (showed in Fig. 2) for each specific area and cause of death for both sexes (5 areas + all Italy, 3 causes + all of them together (DoD) and 2 sex = 48 time-series each with 35 years of observations). We then perform 4 within and 6 between analyses for each sex.

ADF, PP and KPPS tests report that 96.7% of the time series is Non-Stationary (all but PGLEs due to drugs in the Centre for males) with 0.05 p-value, and the 93.3% of the series are integrated of order 1.

Table 2 and Table 3 show all statistically significant relationships reported by Johansen’s approach and whether the relationships found are stationary or not.

Table 2, contains the between-areas analysis results. A long-term equilibrium between PGLE trends obtained by removing all Deaths of Despair among women was revealed, and also by removing only drug-related deaths. A non-stationary relationship, so we cannot state that there is (a long-term) equilibrium, was shown between the trends in PGLE due to alcohol among males.

The within-area analysis (see Table 3) did not reveal any significant dependency structure among the causes of death from despair, even when the significance threshold was relaxed by increasing the p-value cutoffs from 0.05 to 0.1 or 0.25.

**Table 2:** Number of relation found with Johansen’s eigen and trace test for each cause **between** Italian macro areas and whether these relationships are Stationary, meaning the evidence of a long-run equilibrium

		**Number of relations**	
**DoD**	Male	0	
	Female	1	Stationary
**Sucide**	Male	0	
	Female	0	
**Alcohol**	Male	1	Non-Stationary
	Female	0	
**Drugs**	Male	0	
	Female	2	Stationary

**Table 3:** Number of relation found with Johansen’s eigen and trace test **within** Italy and each Italian macro-areas between the three causes together and separately and whether these relationships are Stationary, meaning the evidence of a long-run equilibrium

		**Number of relations**
**Italy**	Male	0
	Female	0
**North-West**	Male	0
	Female	0
**North-East**	Male	0
	Female	0
**Centre**	Male	0
	Female	0
**South**	Male	0
	Female	0
**Islands**	Male	0
	Female	0

#### Short-run equilibrium

As mentioned in the section, we note slight trend breaks in the transition from ICD9 to ICD10 classification for the different causes. We therefore propose the same within and between analyses on the two sub-periods 1983-2002 and 2003-2018.

Generally, the use of cointegration analysis is suggested on extended time series: the larger the sample, the more reliable the evidence. The results reported here are consequently less reliable than those on a long-term basis, but we believe they can help to get a clearer idea of the long-term dynamics. In both sub-periods, more than 90% of the time series are Non-Stationary (95.8% and 93.8% respectively) and the percentage of integrated series of order 1 decreases over the whole period (77.1% and 54.2%).

No short-run equilibrium is shown in the analysis within, confirming that the trend of the three causes at the national level and in the individual macro-areas is completely independent and unrelated. In contrast, we find some equilibrium relationships for individual causes within the macro-areas. The results are summarised in Table 4. Most of the cointegration relationships detected did not turn out to be statistically stationary, so we cannot identify a short- (or long-term) equilibrium in these relationships. On the other hand, the trend in alcohol-related PGLE in the female population between 1983 and 2018 is in equilibrium. Throughout the ICD10 classification, the time series of the drug- and alcohol-related PGLEs of men in the Nuts1 areas are in equilibrium.

**Table 4:** For each multivariate time series is reported if there are **Stationary** cointegrating relationships, *Non-Stationary* relationships or there are *No Relations*

		1983-2002 (ICD9)	2003-2018 (ICD10)
**DoD**	Male	No relations	Non-Stationary
	Female	Non-Stationary	Non-Stationary
**Suicide**	Male	No relations	Non-Stationary
	Female	No relations	Non-Stationary
**Alcohol**	Male	Non-Stationary	**Stationary**
	Female	**Stationary**	Non-Stationary
**Drugs**	Male	No relations	**Stationary**
	Female	No relations	Non-Stationary

Bold values indicate stable equilibrium

The cointegration analysis on the sub-periods confirms the absence of a dependency structure between the 3 causes nationally and within each macro area. At the same time, there are stationary (and non-stationary) cointegration relations between the Nuts1 areas for some of the causes.

## Discussion

### Main findings

We examined the “Deaths of Despair” in Italy, defined as deaths due to alcohol and drug abuse and suicide, using data from 1983 to 2018.

Our analysis found a general decline in the impact of these causes on Italy’s overall mortality, as shown by the *Potential Gain in Life Expectancy* (PGLE) measure. This decline is primarily driven by the decrease in alcohol-related mortality. However, some disparities were noted; for example, alcohol had the highest impact in most years, except following the 2008 economic crisis, when suicides temporarily had a larger effect. Drug-related mortality consistently had the lowest impact, contrasting with trends in the US [15].

Compared to other nations, especially the US, the magnitude of these causes in Italy is relatively lower [33]. In addition, our findings highlight that, while other studies emphasize substantial increases in deaths of despair in several high-income countries [2, 20, 31], our results suggest a more contained but persistent issue within the Italian context. These causes remain significant but are distributed differently across regions and age groups, and are impacted variably by socio-economic crises.

Significant differences emerged across Italy’s regions, particularly between North and South. Alcohol-related mortality generally showed a more prominent decrease in Northern regions, where it reached a lower impact of suicides, especially in the wake of economic crises. Southern regions exhibited slower progress in alcohol-related mortality, which retained a greater impact than suicides. This regional variation may reflect social and economic differences that influence the population’s vulnerability to despair-driven outcomes. The North-South gradient in alcohol and suicide patterns highlights the importance of targeted policy interventions. The high density of small and medium-sized enterprises (SMEs) in the North—Italy’s industrial powerhouse—may have amplified the impact of economic downturns on despair-related deaths, manifested by suicides. Meanwhile, in the South, strong Catholic and traditional values contribute to stigmatizing suicide, potentially shifting despair-driven outcomes toward alcohol-related deaths instead [22]. The North-South gradient in alcohol and suicide patterns highlights the importance of targeted policy interventions.

The results from the Cointegration Analysis underscore that, in Italy, deaths from alcohol, drugs, and suicide cannot be seen as a single, cohesive category. Unlike in some other countries, where these causes may have intertwined patterns, our findings show that these outcomes operate independently over time. This suggests that policies addressing these causes in Italy should consider the unique trends and factors influencing each cause separately rather than approaching them as a single issue. Policies focused on regional disparities are especially critical, as they could enhance the effectiveness of public health interventions aimed at specific causes or regions.

### Study limitations

While comparisons with the US and other countries provide useful context, the Italian situation reflects a fundamentally different profile in both the magnitude and distribution of these causes. Italy has a much lower overall burden of deaths of despair and distinct trends within each cause. This result is in line with what [33] reported. Compared to their findings, however, we observe a slightly higher potential gain in life expectancy (PGLE), likely due to two main reasons. First, their analyses report PGLE at age 25, whereas ours refer to PGLE at birth. We also conducted analyses considering PGLE at ages 25 and 65, and the results do not show significant changes. Second, our slightly higher values stem from the inclusion of K73 and K74 (liver cirrhosis) in our analyses. Although these causes are not entirely attributable to alcohol consumption, we chose to include them to retain a substantial portion of alcohol-related mortality in the model, consistent with approaches used in Southern Europe [41] and in the Anglo-Saxon countries comparison [20]. Indeed, the selection of causes included in the analysis represents one of the limitations of this study.

Another potential limitation lies in our focus on three underlying causes of death, as we prioritized long-term trends in regional mortality. However, despair may not always be recorded as the underlying cause of death but might instead appear elsewhere on the death certificate, particularly in cases related to substance use, such as drugs or alcohol [24, 45]. In this respect, the accuracy with which deaths are certified and coded is fundamental for the validity of our findings. ISTAT data are generally recognized for their high quality and reliability [29], but potential underreporting or misclassification of specific causes (especially suicides, as discussed in Subsection "Main findings") may contribute to regional differences and should be carefully considered when interpreting the results. Moreover, our analyses span a period that includes a change in the ICD classification system. Although we attempted to account for this in Subsection "Short-run equilibrium", it is important to acknowledge that coding transitions may introduce discontinuities in the historical series.

An additional limitation concerns the spatial resolution of our analyses. While the underlying data are available at the provincial (NUTS3) level, we aggregated them to the macro-area (NUTS1) level for both substantive and methodological reasons, as discussed in Sect. "Data". This choice was necessary to reduce small-number volatility particularly for drug-related mortality in narrow age-sex strata—and to provide sufficiently stable series for meaningful comparisons over time. However, this aggregation prevents the study from capturing finer-grained geographical heterogeneity, which could be relevant for identifying localized areas disproportionately affected by despair-related deaths.

Finally, the study is limited to data up to 2018, which prevents us from capturing more recent developments in mortality trends related to despair. This temporal restriction also highlights the importance of extending the analysis to incorporate more recent data, including the period of the COVID-19 pandemic, as emerging evidence suggests it may have exacerbated despair-related mortality [3].

### Conclusion

Our findings support the need to analyse these causes separately in Italy rather than aggregating them under the term Deaths of Despair. With the absence of structural dependence among the causes, we argue that these represent distinct aspects of societal despair, manifesting differently by age and region. These results suggest that the US-specific DoD pattern does not translate directly into the Italian context. This finding should be interpreted in light of the original formulation of the DoD thesis [14–16], which, as discussed in Sect. "State of art on Deaths of Despair", referred to a specific demographic subgroup—white non-Hispanic, middle-aged individuals with low educational attainment—within the United States. The lack of convergence in trends at the aggregate Italian level does not preclude the possibility that some Italian subpopulations, defined for example by sex, education, ethnicity, or region, could exhibit mortality patterns more consistent with the DoD hypothesis. However, the data employed in this study do not include additional socio-economic information on the deceased, making further subgroup analyses unfeasible. Future research that integrates cause-specific mortality data with detailed socio-demographic information would be necessary to explore such within-country heterogeneity and assess whether the phenomenon might emerge in specific segments of the Italian population. Moreover, by exploiting different analytical approaches, future studies could allow for a more detailed investigation at finer geographic scales. Such analyses would provide valuable insights into the spatial magnitude of despair-related mortality, complementing the temporal focus adopted in this first study of the Italian context. Finally, it will be necessary to examine more recent years, including those encompassing the COVID-19 pandemic.

Although the Italian experience appears less severe than that observed in the US or other contexts, the impact remains non-negligible: in 2018, deaths of despair accounted for a cumulative loss in life expectancy of nearly five months.

## Figures and Tables

**Fig. 3:**
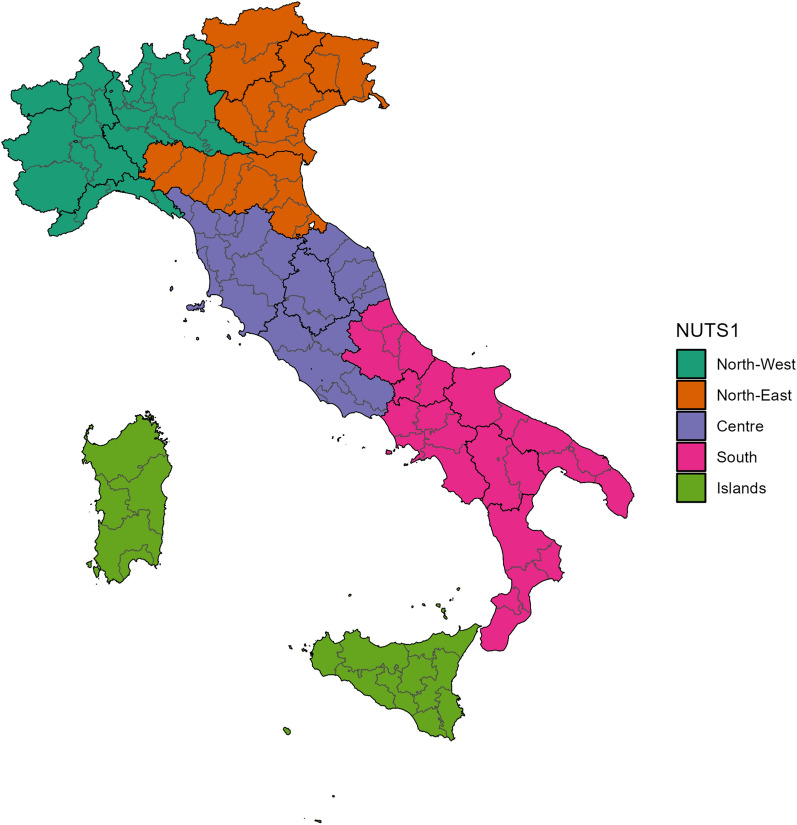
Map of Italian regions grouped into macroareas (NUTS1). Colors indicate the macroarea classification: North-West, North-East, Centre, South, and Islands

**Fig. 6:**
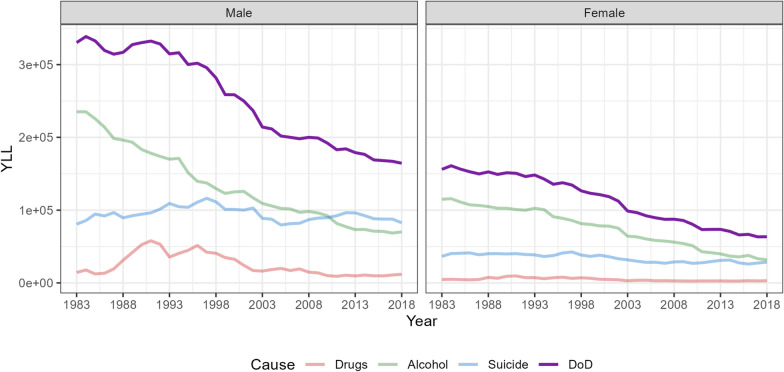
Years of Life Lost removing Drugs, Alcohol and Suicide separately or together (DoD, violet line). Italy 1983-2018

**Fig. 7:**
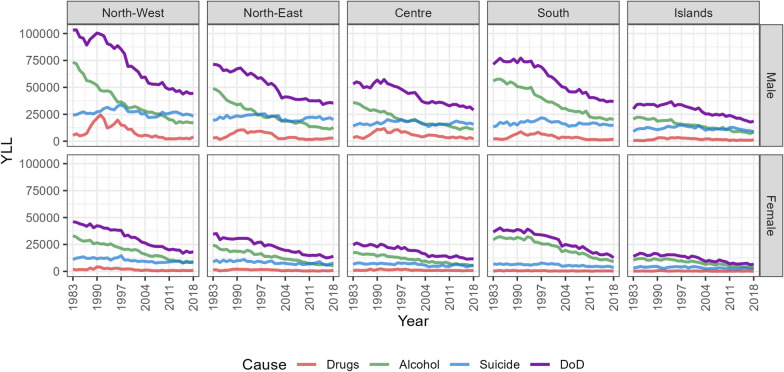
Years of Life Lost removing Drugs, Alcohol and Suicide separately or together (DoD, violet line). Nuts1 1983-2018

## Data Availability

The data that support the findings of this study are available from ISTAT (Italian National Institute of Statistics) but restrictions apply to the availability of these data, which were used under license for the current study, and so are not publicly available. Data are however available from the authors upon reasonable request and with permission of ISTAT (Italian National Institute of Statistics).

## A Italian macroareas: Nuts1

See Fig. 3

## B Age-at-Death of Despair distribution

The age-at-death distribution varies between the three causes, between sexes and across time.

Looking at Fig. 4 we can see that the modal age-at-death related to drug abuse shifted on the left over time. For both males and females, there is a peak in the frequency of drug-related deaths in the 1990s. At higher ages there is an increase in the curve for females, we assume this is mainly due to deaths related to mental and behavioural disorders due to use of drugs and accidental poisoning due to poorly recorded medical treatment. In any case, the numbers are small and do not interfere with the analysis carried out.

Alcohol-related deaths also have a modal age moving towards older ages over the years, especially among men marking a convergence with the modal age at death from alcohol for women, but maintaining higher variability.

Suicide has a bimodal distribution. The first of the two modes (the one around age 50) increased for males and females over time. Of the three causes, suicides have the greatest variability in the age-at-death.

## C Potential gain in life expectancy at nuts1 level

The measure of Potential Life Expectancy Gain is a measure that can be compared between different regions, different years, different genders, and different causes of death. The measure of Potential Life Expectancy Gain is a measure that can be compared between different regions, different years, different genders and different causes of death. Fig. 5 shows all the calculated values in a single scale, so that comparisons can be made concerning each dimension.

Most of the comments were reported in Sect. "Cointegration analysis between and within areas". Here we would like to highlight just a few additional aspects. The Centre reports the darkest colours in the drug-related PGLE, for both males and females. The South stands out in alcohol-related mortality and the North-East and Islands in Suicide-related heat maps. The North-West maintains medium-high levels in each of the causes.

## D years of life lost

### D.1 years of life lost (YLL)

The *Years of Life Lost* (YLL) is a widely used indicator for quantifying premature mortality [36]. Conceptually, YLL measures the gap between the actual age at death and the expected remaining lifetime according to a standard reference life table [35]. It therefore captures the total number of years of potential life lost due to deaths occurring before the end of a full lifespan.

Formally, for cause $C$, let $D_x^C$ denote the number of deaths at age $x$ due to cause $C$, and let $e_x^{std}$ be the remaining life expectancy at age $x$ from a standard life table. In our case, the standard life table is the complete life table for each geographical area. The YLL for cause $C$ is defined as:

$$YLL^C = \sum _x D_x^C \cdot e_x$$

This definition implies that deaths at younger ages contribute more heavily to YLL than those at older ages, since the remaining life expectancy is larger at younger ages.

### D.2 results adopting years of life lost

We report here the Years of Life Lost (YLL) results in parallel to the PGLE results presented in Sect. "Results" of the main manuscript. Figure 6 shows the national-level YLL time series for males and females, while Fig. 7 presents the corresponding series by macro-area (NUTS1).

At the national level, the total YLL attributable to the three causes combined decreased over the observation period. In the first year analysed the years of life lost due to the three causes jointly amounted to approximately 330,000 for men and were substantially lower for women; by 2018 the combined YLL had fallen to 164,426 for men and 63,366 for women. The temporal pattern closely mirrors the PGLE evidence: most of the reduction in premature mortality is explained by a decline in alcohol-related YLL, and after the period of economic strain suicides became relatively more important than alcohol in shaping the male time series.

At the macro-area level the YLL trajectories reproduce the general picture observed with PGLE: the same broad regional patterns are visible across the five NUTS1 areas, and no macro-area displays radically different dynamics for any of the three causes. The similarity between Figures 7 and the PGLE heatmaps indicates that both indicators capture the same essential temporal and spatial signals, with only nuanced differences in relative magnitudes across sexes and areas.

We also applied the same cointegration workflow to the YLL series in order to test for long-run equilibria across macro-areas and among the three causes within each area. Results are summarised in Tables 5 and 6. These findings largely replicate the cointegration outcomes obtained from the PGLE series (see Tables 2 and 3 in the main text): in nearly all cases the presence or absence of long-run relationships is concordant across the two metrics. The single notable exception is an additional stationary cointegrating relation detected for female alcohol-related YLL across regions; otherwise the YLL-based tests confirm the PGLE conclusion that alcohol-, drug- and suicide-related mortality do not generally follow a single, common long-run trend at the national or macro-area level in Italy.

**Table 5:** Number of relation found with Johansen’s eigen and trace test for each cause **between** Italian macro areas and whether these relationships are Stationary, meaning the evidence of a long-run equilibrium

		**Number of relations**	
**DoD**	Male	0	
	Female	1	Stationary
**Sucide**	Male	0	
	Female	0	
**Alcohol**	Male	1	Non-Stationary
	Female	2	Stationary
**Drugs**	Male	0	
	Female	2	Stationary

**Table 6:** Number of relation found with Johansen’s eigen and trace test **within** Italy and each Italian macro-areas between the three causes together and separately and whether these relationships are Stationary, meaning the evidence of a long-run equilibrium

		**Number of relations**
**Italy**	Male	0
	Female	0
**North-West**	Male	0
	Female	0
**North-East**	Male	0
	Female	0
**Centre**	Male	0
	Female	0
**South**	Male	0
	Female	0
**Islands**	Male	0
	Female	0

Taken together, the parallel results obtained using an alternative mortality impact measure reinforce the robustness of our main findings. The agreement between PGLE and YLL — two conceptually related but distinct indicators — increases confidence that the observed lack of structural dependence among the three causes is not an artefact of the chosen metric but a consistent feature of the Italian data.
